# Progress Toward Polio Eradication — Worldwide, January 2019–June 2021

**DOI:** 10.15585/mmwr.mm7034a1

**Published:** 2021-08-27

**Authors:** John Paul Bigouette, Amanda L. Wilkinson, Graham Tallis, Cara C. Burns, Steven G. F. Wassilak, John F. Vertefeuille

**Affiliations:** ^1^Epidemic Intelligence Service, CDC; ^2^Global Immunization Division, Center for Global Health, CDC; ^3^Polio Eradication Department, World Health Organization, Geneva, Switzerland; ^4^Division of Viral Diseases, National Center for Immunization and Respiratory Diseases, CDC.

In 1988, when the Global Polio Eradication Initiative (GPEI) began, polio paralyzed >350,000 children across 125 countries. Today, only one of three wild poliovirus serotypes, type 1 (WPV1), remains in circulation in only two countries, Afghanistan and Pakistan. This report summarizes progress toward global polio eradication during January 1, 2019–June 30, 2021 and updates previous reports (*1*,*2*). In 2020, 140 cases of WPV1 were reported, including 56 in Afghanistan (a 93% increase from 29 cases in 2019) and 84 in Pakistan (a 43% decrease from 147 cases in 2019). As GPEI focuses on the last endemic WPV reservoirs, poliomyelitis outbreaks caused by circulating vaccine-derived poliovirus (cVDPV) have emerged as a result of attenuated oral poliovirus vaccine (OPV) virus regaining neurovirulence after prolonged circulation in underimmunized populations (*3*). In 2020, 32 countries reported cVDPV outbreaks (four type 1 [cVDPV1], 26 type 2 [cVDPV2] and two with outbreaks of both); 13 of these countries reported new outbreaks. The updated GPEI Polio Eradication Strategy 2022–2026 (*4*) includes expanded use of the type 2 novel oral poliovirus vaccine (nOPV2) to avoid new emergences of cVDPV2 during outbreak responses (*3*). The new strategy deploys other tactics, such as increased national accountability, and focused investments for overcoming the remaining barriers to eradication, including program disruptions and setbacks caused by the COVID-19 pandemic.

## Polio Vaccination

In worldwide immunization programs, OPV and at least 1 dose of injectable, inactivated poliovirus vaccine (IPV) are routinely used. Because IPV contains all three poliovirus serotypes, it protects against disease in children who seroconvert after vaccination; however, it does not prevent poliovirus transmission. In 2016, a global coordinated switch occurred from trivalent OPV (tOPV), which contains Sabin strain types 1, 2, and 3 to bivalent OPV (bOPV), which contains Sabin strain types 1 and 3. WPV2 was declared eradicated in 2015, and cVDPV2 was the predominant cause of cVDPV outbreaks after the last WPV2 case was detected in 1999. The use of monovalent OPV Sabin strain type 2 (mOPV2) is reserved for cVDPV2 outbreak responses. In November 2020, the World Health Organization (WHO) granted Emergency Use Listing (EUL) for genetically stabilized nOPV2 to be used in a limited number of countries that have met readiness criteria for initial use* of nOPV2 (*5*) in response to outbreaks.

In 2020, the estimated global infant coverage with 3 doses of poliovirus vaccine (Pol3) by age 1 year was 83% (*6*). However, substantial variation in coverage exists by WHO region, nationally, and subnationally. In the two countries with endemic WPV (Afghanistan and Pakistan), 2020 POL3 coverage was 75% and 83%, respectively (*6*); estimated coverage in subnational areas with transmission is much lower.

In 2019, GPEI supported 199 supplementary immunization activities (SIAs)^†^ in 42 countries with approximately 1 billion bOPV, 20 million IPV, 32 million monovalent OPV type 1 (mOPV1), and 142 million mOPV2 doses administered. In 2020, 149 SIAs were conducted in 30 countries with approximately 696 million bOPV, 6 million IPV, 4 million mOPV1, 228 million mOPV2, and 51 million tOPV doses administered; tOPV was used during four SIAs in Afghanistan and Pakistan, where cocirculation of WPV1 and cVDPV2 requires tOPV for efficiency in scheduling and implementing SIAs; GPEI authorized restarting filling of tOPV stocks for this purpose. In 2021, approximately 136 million nOPV2 doses have been released in eight countries approved for initial use (Benin, Chad, Congo, Liberia, Niger, Nigeria, Sierra Leone, and Tajikistan). SIAs continue to be affected by the COVID-19 pandemic^§^ in 2021.

## Poliovirus Surveillance

WPV and cVDPV transmission are detected primarily through surveillance for acute flaccid paralysis (AFP) among children aged <15 years with testing of stool specimens at one of 145 WHO-accredited laboratories of the Global Polio Laboratory Network (*7*). During January–September 2020, the number of reported AFP cases declined 33% compared with the same period in 2019 (*8*). Environmental surveillance (testing of sewage for poliovirus) can supplement AFP surveillance; however, environmental sampling also declined somewhat during this period. Current data indicate that the COVID-19 pandemic has continued to limit AFP surveillance sensitivity. The continued strengthening of both surveillance systems, particularly in priority countries,^¶^ is critical to tracking progress and documenting the absence of poliovirus transmission.

## Reported Poliovirus Cases and Isolations

**Countries reporting WPV cases and isolations.** Since 2016, no WPV cases have been identified outside of Afghanistan and Pakistan. Of the 176 WPV1 cases reported in 2019, 29 (16%) occurred in Afghanistan and 147 (84%) in Pakistan (Figure) (Table 1).

**FIGURE:**
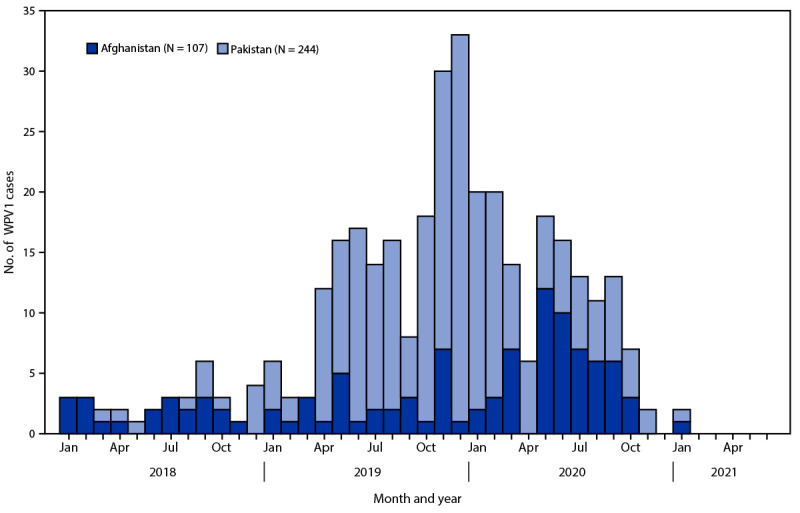
Number of wild poliovirus type 1 cases, by country and month of paralysis onset — worldwide, January 2019—June 2021* **Abbreviation:** WPV1 = wild poliovirus type 1. * Data are current as of August 3, 2021.

**TABLE 1:** Number of poliovirus cases, by country — worldwide, January 1, 2019–June 30, 2021*

Country (cVDPV type)	Reporting period							
	2019		2020		Jan–Jun 2020		Jan–Jun 2021	
	WPV1	cVDPV	WPV1	cVDPV	WPV1	cVDPV	WPV1	cVDPV
**Countries with endemic WPV1 transmission**								
Afghanistan (2)^†^	29	0	56	308	34	54	1	43
Pakistan (2)	147	22	84	135	60	52	1	8
**Countries with reported cVDPV cases**								
Angola (2)	0	138	0	3	0	3	0	0
Benin (2)	0	8	0	3	0	2	0	2
Burkina Faso (2)	0	1	0	65	0	26	0	1
Burma (Myanmar)(2)^§^	0	6	0	0	0	0	0	0
Cameroon (2)^†^	0	0	0	7	0	4	0	0
Central African Republic (2)	0	21	0	4	0	1	0	0
Chad (2)	0	11	0	99	0	57	0	0
China (2)	0	1	0	0	0	0	0	0
Republic of the Congo (2)^†^	0	0	0	2	0	0	0	2
Côte d’Ivoire (2)^†^	0	0	0	61	0	39	0	0
Democratic Republic of the Congo (2)	0	88	0	81	0	54	0	10
Ethiopia (2)	0	14	0	36	0	17	0	6
Ghana (2)	0	18	0	12	0	12	0	0
Guinea (2)^†^	0	0	0	44	0	23	0	6
Liberia (2)^†^	0	0	0	0	0	0	0	3
Madagascar (1)^†^	0	0	0	2	0	0	0	6
Malaysia (1)	0	3	0	1	0	1	0	0
Mali (2)^†^	0	0	0	48	0	6	0	0
Niger (2)	0	1	0	10	0	6	0	0
Nigeria (2)	0	18	0	8	0	2	0	65
Philippines (1,2)^¶^	0	14	0	1	0	1	0	0
Senegal (2)^†^	0	0	0	0	0	0	0	13
Sierra Leone (2)^†^	0	0	0	10	0	0	0	4
Somalia (2)	0	3	0	14	0	2	0	0
South Sudan (2)^†^	0	0	0	50	0	2	0	9
Sudan (2)^†^	0	0	0	58	0	10	0	0
Tajikistan (2)^†^	0	0	0	1	0	0	0	23
Togo (2)	0	8	0	9	0	9	0	0
Yemen (1)	0	1	0	31	0	22	0	3
Zambia (2)	0	2	0	0	0	0	0	0

**Abbreviations:** cVDPV = circulating vaccine-derived poliovirus; WPV1 = Wild poliovirus type 1.

* Data are current as of August 3, 2021.

^†^ New cVDPV cases reported after December 31, 2019.

^§^ For this country, *MMWR* uses the U.S. State Department short-form name “Burma”; the World Health Organization uses “Myanmar.”

^¶^ Reported two cVDPV type 1 cases and 12 cVDPV type 2 cases in 2019, one cVDPV type 2 case in 2020.

In 2020, Afghanistan reported 56 WPV1 cases representing a 93% increase from cases reported in the previous year; cases were found across 38 districts compared with 20 districts in 2019. As of August 3, 2021, one WPV1 case was reported in Afghanistan in 2021, a 97% decrease compared with the first 6 months of 2020. Pakistan reported 84 WPV1 cases from 39 districts in 2020, representing a 43% decrease from the 147 cases reported in 43 districts during 2019. One WPV1 case has been reported during January–June 2021, from Balochistan province, a 98% decrease from the 60 WPV1 cases from five provinces during the same 2020 period. This period accounted for 71% of all Pakistan WPV1 cases in 2020. In both countries, the number of orphan WPV1 isolates (those with ≤98.5% genetic identity with other isolates) from AFP cases increased from five of 176 (3%) in 2019 to 18 of 140 (13%) in 2020, signifying an increase in AFP surveillance gaps in 2020 (*7*).

Environmental surveillance in Afghanistan detected WPV1 in 35 (8%) of 418 sewage samples collected during 2020 and in 57 (22%) of 264 samples in 2019 (Table 2). In Pakistan, WPV1 was detected in 434 (52%) of 830 sewage samples collected in 2020, and 44% (379/854) of sewage samples were WPV1-positive in 2019. In 2019, three (4%) of the 71 sewage samples collected in Iran contained WPV1 isolates; no positive environmental samples or cases have been reported since then.

**TABLE 2:** Number of circulating wild polioviruses and circulating vaccine-derived polioviruses detected through environmental surveillance — worldwide, January 1, 2019–June 30, 2021*

Country	Jan 1–Dec 31, 2019		Jan 1–Dec 31, 2020		Jan 1–Jun 30, 2020		Jan 1–Jun 30, 2021	
	No. of samples	No. (%) with isolates	No. of samples	No. (%) with isolates	No. of samples	No. (%) with isolates	No. of samples	No. (%) with isolates
**Countries with reported WPV1-positive samples (no. and percentage of isolates refer to WPV1)**								
Afghanistan	264	57 (22)	418	35 (8)	172	22 (13)	213	1 (1)
Iran	71	3 (4)	43	0 (—)	0	0 (—)	0	0 (—)
Pakistan	854	379 (44)	830	434 (52)	414	238 (57)	444	59 (13)
**Countries with reported cVDPV-positive samples (cVDPV type) (no. and percentage of isolates refer to cVDPVs)**								
Afghanistan (2)	264	0 (—)	418	175 (42)	172	46 (27)	213	40 (19)
Angola (2)	106	17 (16)	98	0 (—)	47	0 (—)	15	0 (—)
Benin (2)	37	0 (—)	70	5 (7)	31	0 (—)	52	1 (2)
Cameroon (2)	602	4 (1)	273	9 (3)	134	4 (3)	187	0 (—)
Central African Republic (2)	149	10 (7)	97	2 (2)	43	2 (5)	48	0 (—)
Chad (2)	198	10 (5)	77	3 (4)	55	3 (5)	26	0 (—)
China (3)	0	0 (—)	0	0 (—)	0	0 (—)	1	1 (100)
Republic of the Congo (2)	0	0 (—)	12	1 (8)	0	0 (—)	213	1 (1)
Cote d’Ivoire (2)	154	7 (5)	130	91 (70)	88	62 (70)	42	0 (—)
Democratic Republic of the Congo (2)	294	0 (—)	170	1 (1)	78	1 (1)	145	0 (—)
Egypt (2)	703	0 (—)	550	1 (0)	267	0 (—)	313	10 (3)
Ethiopia (2)	159	3 (2)	51	2 (4)	33	0 (—)	15	0 (—)
Gambia (2)	0	0 (—)	0	0 (—)	0	0 (—)	9	2 (22)
Ghana (2)	202	17 (8)	184	20 (11)	100	19 (19)	99	0 (—)
Guinea (2)	103	0 (—)	67	1 (1)	38	0 (—)	61	0 (—)
Iran (2)	74	0 (—)	43	3 (7)	12	0 (—)	25	1 (4)
Kenya (2)	317	0 (—)	193	1 (1)	92	0 (—)	101	1 (1)
Liberia (2)	0	0 (—)	34	6 (18)	15	0 (—)	47	12 (26)
Madagascar (1)	520	0 (—)	351	0 (—)	232	0 (—)	134	12(9)
Malaysia (1, 2)	13	12 (92)	76	12 (16)	50	12 (24)	22	0 (—)
Mali (2)	48	0 (—)	44	4 (9)	22	2 (9)	27	0 (—)
Niger (2)	293	0 (—)	157	7 (4)	93	1 (1)	73	0 (—)
Nigeria (2)	2071	60 (3)	1294	5 (0)	625	0 (—)	868	34 (4)
Pakistan (2)	855	36 (4)	830	135 (16)	414	35 (8)	444	32 (7)
Philippines (1, 2)	67	32 (48)	80	4 (5)	50	4 (8)	18	0 (—)
Senegal (2)	56	0 (—)	27	1 (4)	14	0 (—)	10	4 (40)
Somalia (2)	92	5 (5)	88	26 (30)	52	18 (35)	52	1 (2)
South Sudan (2)	111	0 (—)	85	6 (7)	57	0 (—)	24	0 (—)
Sudan (2)	65	0 (—)	50	14 (28)	20	3 (15)	30	0 (—)
Tajikistan (2)	0	0 (—)	0	0 (—)	0	0 (—)	14	13 (93)
Uganda (2)	56	0 (—)	58	0 (—)	24	0 (—)	36	2 (6)

**Abbreviations:** cVDPV = circulating vaccine-derived poliovirus; WPV1 = Wild poliovirus type 1.

* Data are current as of August 3, 2021.

**Countries reporting cVDPV cases and isolations.** During January 2019–June 2021, cVDPV transmission was identified in 32 countries; 13 countries were affected by new cVDPV outbreaks in 2020. Afghanistan reported 308 cVDPV2 cases in 2020 compared with no cases in 2019. Pakistan reported 135 cVDPV2 cases in 2020, more than a fivefold increase from the 22 reported in 2019. To date in 2021, 195 cVDPV2 cases have been identified globally, including 43 in Afghanistan and eight in Pakistan.

## Discussion

With the August 2020 certification of the African Region as WPV-free,** five of the six WHO regions, representing over 90% of the world’s population, are now free of wild polioviruses. Given this achievement, GPEI is focusing efforts on two goals: interrupting persistent WPV1 transmission in Pakistan and Afghanistan and stopping all current outbreaks of cVDPV2. To reach these goals, in June 2021, GPEI released a revised 5-year strategy for polio eradication that aims to address persistent challenges and recover from setbacks exacerbated by the COVID-19 pandemic (*4*).

Guided by the Polio Eradication Strategy 2022–2026, GPEI partners and in-country stakeholders are to adopt a full emergency posture and assume more accountability for eradication at every level of the program (*4*). The strategy elevates efforts in the highest-risk countries and promotes health service integration, surveillance improvement, and community engagement to enhance campaign quality through increased political advocacy to ensure timely and effective emergency outbreak SIA responses through improved government support of implementation.

Although Pakistan and Afghanistan face distinct challenges, they are linked epidemiologically because of high rates of cross-border population movement. Transit-point vaccination must be maintained as emigration from Afghanistan potentially increases in 2021. The beginning of each year is typically the low season for WPV1 transmission in both countries, and AFP surveillance sensitivity has decreased. During 2019, the Pakistan polio program suffered from increased vaccine resistance fed by social media misinformation and faced continued operational problems in some localities. The program changed its management oversight and enhanced efforts to overcome community mistrust to decrease vaccine hesitancy (*9*). Inroads to improving the effectiveness of the SIAs have also been made in 2020 (*4*). Although the proportion of Pakistan environmental samples that are WPV-positive remains high in 2021 to date, the decrease from the same period in 2020 is worth noting.

In Afghanistan, the main challenges to ending poliovirus transmission are the inability to reach all children in critical areas near reservoirs in Pakistan and increasing political instability. The polio program in Afghanistan has continued to operate for many years, even during periods of insecurity and escalating conflict. Although negotiations with local leaders in Afghanistan facilitated vaccination efforts at one time, restrictions on vaccinations have persisted in areas controlled by insurgent groups since the October 2018 ban on house-to-house campaigns, which has since expanded geographically (*10*). WHO is anticipating that some negotiated access will again be possible. Other challenges include current mass population movements, clusters of vaccine refusals, and suboptimal SIA quality in some areas previously under government control (*10*).

Globally, cVDPV2 outbreaks increased in number and geographic extent during 2019–2020 because of delays in mOPV2 response SIAs, which were frequently of low quality. Since the switch in 2016 from tOPV to bOPV, 1,755 cases of paralytic polio have been reported from 64 cVDPV2 outbreaks in 30 countries across four WHO regions (*4*).^††^ GPEI has outlined a strategy for stopping cVDPV transmission and reducing the risk of seeding new outbreaks by expanding use of nOPV2 (*4*). Continued monitoring will be needed to ensure safety and effectiveness while nOPV2 is brought into wider use and to ascertain whether it can replace mOPV2 (*5*).

The findings in this report are subject to at least one limitation. SIAs, field surveillance, and investigation activities were curtailed in 2020 because of COVID-19 pandemic mitigation measures, and laboratory testing suffered delays (*8*); limitations on SIA quality and surveillance sensitivity continue in 2021. On the other hand, the COVID-19 pandemic has presented opportunities to jointly increase the effectiveness of polio eradication activities and promote health services integration. For example, the global rollout of COVID-19 vaccines presents an opportunity to strengthen demand for vaccination against both COVID-19 and polio. 

Thousands of polio eradication workers worldwide continue to play a critical role in implementing countries’ COVID-19 responses. Maintaining these partnerships will be important in eradicating WPV and stopping cVDPV transmission while simultaneously addressing other health priorities.

**Summary**

**What is already known about this topic?**

Wild poliovirus type 1 (WPV1) remains endemic in Afghanistan and Pakistan. Circulating vaccine-derived poliovirus type 2 (cVDPV2) outbreaks have increased since 2017.

**What is added by this report?**

From 2019 to 2020, the number of WPV1 cases increased in Afghanistan and decreased in Pakistan and the number of cVDPV2 cases increased and cVDPV2 outbreak countries increased to 32. In Afghanistan, the polio program faces challenges including an inability to reach children in critical areas and increasing political instability. The COVID-19 pandemic continues to limit the quality of immunization activities and poliovirus surveillance.

**What are the implications for public health practice?**

The Polio Eradication Strategy for 2022–2026 outlines measures including increased government accountability and wider use of novel, oral poliovirus vaccine type 2 that are needed to eradicate polio.

## Footnotes

* Authorization for wider use under EUL is pending review of safety and effectiveness data from the initial use.

† Mass immunization campaigns intended to interrupt poliovirus circulation by immunizing every child aged <5 years with 2 OPV doses, regardless of previous immunization status.

§ GPEI implemented a pause on SIAs from mid-March through July 2020.

¶ 2020 priority countries: *African Region*: Angola, Benin, Burkina Faso, Cameroon, Central African Republic, Chad, Congo, Côte d’Ivoire, Democratic Republic of the Congo, Eritrea, Ethiopia, Ghana, Guinea, Kenya, Liberia, Madagascar, Mali, Mauritania, Mozambique, Niger, Nigeria, Senegal, Sierra Leone, South Sudan, Togo, and Zambia; *Eastern Mediterranean Region*: Afghanistan, Egypt, Iran, Iraq, Libya, Pakistan, Somalia, Sudan, Syria, and Yemen; *European Region*: Tajikistan and Uzbekistan; *South-East Asia Region*: Burma (Myanmar); *Western Pacific Region*: Malaysia and Philippines.

**
https://www.africakicksoutwildpolio.com

††
https://polioeradication.org/polio-today/polio-now/this-week/circulating-vaccine-derived-poliovirus/
